# Multifunctional Iron Oxide Nanoparticles as Promising Magnetic Biomaterials in Drug Delivery: A Review

**DOI:** 10.3390/jfb15080227

**Published:** 2024-08-14

**Authors:** Katja Vasić, Željko Knez, Maja Leitgeb

**Affiliations:** 1 Laboratory for Separation Processes and Product Design, Faculty of Chemistry and Chemical Engineering, University of Maribor, Smetanova ulica 17, 2000 Maribor, Slovenia; katja.vasic@um.si (K.V.); zeljko.knez@um.si (Ž.K.); 2 Faculty of Medicine, University of Maribor, Taborska ulica 8, 2000 Maribor, Slovenia

**Keywords:** multifunctional biomaterials, bio-composites, nanocarriers, magnetic nanoparticles, modification, drug delivery

## Abstract

A wide range of applications using functionalized magnetic nanoparticles (MNPs) in biomedical applications, such as in biomedicine as well as in biotechnology, have been extensively expanding over the last years. Their potential is tremendous in delivery and targeting systems due to their advantages in biosubstance binding. By applying magnetic materials-based biomaterials to different organic polymers, highly advanced multifunctional bio-composites with high specificity, efficiency, and optimal bioavailability are designed and implemented in various bio-applications. In modern drug delivery, the importance of a successful therapy depends on the proper targeting of loaded bioactive components to specific sites in the body. MNPs are nanocarrier-based systems that are magnetically guided to specific regions using an external magnetic field. Therefore, MNPs are an excellent tool for different biomedical applications, in the form of imaging agents, sensors, drug delivery targets/vehicles, and diagnostic tools in managing disease therapy. A great contribution was made to improve engineering skills in surgical diagnosis, therapy, and treatment, while the advantages and applicability of MNPs have opened up a large scope of studies. This review highlights MNPs and their synthesis strategies, followed by surface functionalization techniques, which makes them promising magnetic biomaterials in biomedicine, with special emphasis on drug delivery. Mechanism of the delivery system with key factors affecting the drug delivery efficiency using MNPs are discussed, considering their toxicity and limitations as well.

## 1. Introduction

In recent years, novel intelligent drug delivery systems have played an important role in the biomedical field, especially by implementing functional biomaterials based on different polymers for the development of new smart strategies. As a result, highly advanced multifunctional bio-composites with high specificity, efficiency, and optimal bioavailability are designed and implemented in various bio-applications. One of such bio-composites are magnetic nanoparticle (MNP)-based nanomaterials. 

**Figure 1:**
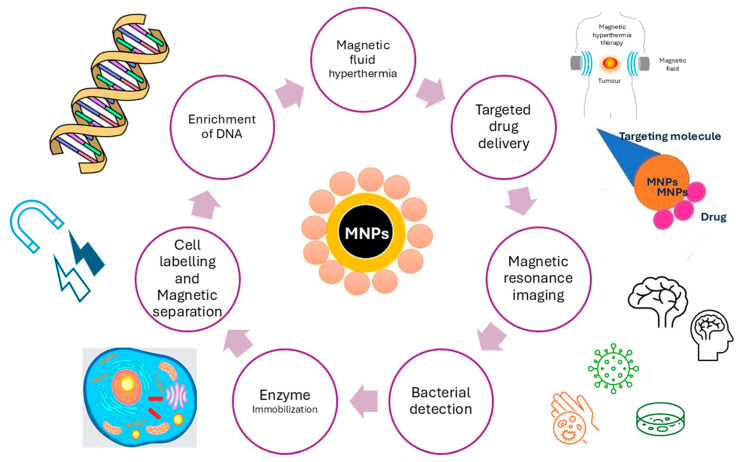
Various biomedical applications of MNPs.

Functionalized magnetic nanocomposites based on iron oxide have been extensively used in biomedical applications due to their significance in several applications, such as targeted drug delivery, cancer treatment, and MRI imaging. Nanotechnology is currently one of the leading areas in applying such magnetic nanocomposites in various applications because of their enormous technological advancements, especially in chemistry, medicine, biology, and pharmacy [1,2]. In general, nanotechnology is covering materials in the size range of 1–100 nm, which implies to one billionth of a meter (10^−9^). In recent years, nanotechnology has gained immense advantages in drug delivery. Especially, MNPs are becoming materials that are extremely popular to be used as nanocarriers with their many advantages, such as ease of preparation, tolerability, biocompatibility, flexibility in accommodating different drugs and bioactive molecules, their control of drug release, high drug accumulation in targeting tissues, and many more [3]. However, many of these nano-systems have low targeting ability, limited permeability, poor drug loading capacity, and physical stability, as well as degradation and toxicity-associated problems. Therefore, it is important to develop and explore new strategies and synthesis protocols to achieve effective and advanced nanocarrier-based medicine. Limited effectiveness, poor distribution, and lack of selectivity are the main characteristics of conventionally marketed medicines, which include anti-cancer medicines [4]. General cancer therapy guidelines include chemotherapy, surgery, and radiation. However, they remain time-limited and poorly curative despite various therapeutic options. Since cancer is the leading cause of deaths in both developed and developing countries, new superior and improved medicines are on the rise and have been a global pursuit in the last decade [5,6,7,8,9,10]. Some of the MNP applications in biomedicine are overviewed in Figure 1.

MNPs have potential for numerous technological applications, which can be applied in nanoscience. Their properties include flexibility, can be modularly designed, and most importantly, have low toxicity [11,12,13,14]. MNPs engineering is generating excellent platforms for biomedical research, where drug delivery systems with the ability of magnetic control are used. Such applications include biosensors and contrast agents for diagnosis [15], as well as in environmental sciences and green chemistry with environmentally friendly approaches [16]. The chemistry of MNPs under an external magnetic field makes them efficient nanocarriers in magnetic separation for different bio-substances or important components, such as proteins, ions, and inorganic or organic pollutants. They can also be used as reusable nanocarriers for biocatalysis, such as magnetic nanocarriers for enzyme immobilization [17,18,19]. The characteristic properties, such as magnetization, surface area, and microstructure, of these materials largely depend on their bulk materials. All characteristics of nanomaterials are primarily determined by their shape, surface, and final size. The shape is mostly related to the shape and geometries of specific composites, which can be cubic, spherical, or shell–core structures, while the surface of composites relates to the presence of different materials bound to the surface. This includes polymer coatings as well as the incorporation of different metals, carbon-based materials, and rare elements. Modification with such materials can prevent agglomeration and aggregation and also enhance the magnetic properties of different composites. The size of composites relates to their size, hydrodynamic diameter, and size distributions. On the other hand, the high surface energy and dipole interactions can lead to excessive aggregation and agglomeration when the composites are not modified with suitable coatings [20].

In this review paper, we present a comprehensive overview of the physical and morphological characteristics of MNPs that influence their applications in biological environments, as well as examine the various applications of MNPs in drug delivery systems. We explore how the design elements of MNPs, including their size, shape, composition, and surface chemistry, enable the creation of magnetic biomaterials as versatile and multifunctional magnetic platforms. Additionally, we provide a critical analysis of the essential properties of MNPs, their surface chemistry, and the conjugation methods employed in various bio-applications.

## 2. Synthesis Strategies of MNPs

**Figure 2:**
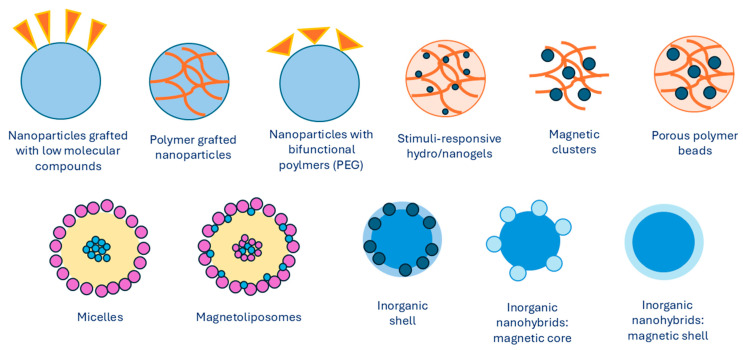
Types of MNPs modified with organic or inorganic polymers. Summarized from [32].

Over the last decades, there have been numerous investigations into preparing MNPs with unique properties and characteristics. The magnetic properties of iron oxide nanoparticles depend on their composition and morphology. Thus, the synthesis methods need to be selected in a way to ensure control over the size, shape, size distribution, and crystallinity of MNPs. Different physical and chemical methods can be used to successfully synthesize iron MNPs. The synthesis of the magnet is the core of a magnetic nanocarrier approach. It is for this reason that many strategies have been developed in order to create a magnetic core that is distinguishable. Excellent superparamagnetic properties, biocompatibility, and biodegradability of iron oxide make it the most widely used magnetic core. In general, MNPs are synthesized using chemical, physical, or microbial methods, with chemical methods being the most common. The most frequently used chemical methods of synthesis involve co-precipitation of iron salts that contain Fe^2+^ and Fe^3+^ ions at basic conditions [21]. Iron oxides, such as Fe_3_O_4_, γ-Fe_2_O_3_, and α-Fe_2_O_3_, can be synthesized by adjusting synthesis conditions. Precipitating an aqueous solution of Fe^2+^)/Fe^3+^ ions or generating Fe^3+^ ions in situ leads to the formation of Fe_3_O_4_ and γ-Fe_2_O_3_ nanoparticles. In contrast, synthesis at higher temperatures yields α-Fe_2_O_3_ nanoparticles. Ferromagnetic nanoparticles can also be produced by first synthesizing hematite, which is more stable, and then reducing it to magnetite. α-Fe_2_O_3_ nanoparticles, known for their antiferromagnetic properties, also exhibit excellent semiconducting behavior. They are more stable, highly resistant to corrosion, and nontoxic, making them suitable for technological applications [22,23]. Some other methods include synthesis and formation of MNPs inside micelles [24], thermal decomposition with organic solvents [25,26,27], sol–gel methods [28,29], electrochemical deposition [30], and hydrothermal synthesis [31]. An overview of different types of MNPs with various core/shell arrangements is presented in Figure 2, which shows the development of MNP design. 

Mentioned synthesis methods produce MNPs with sizes varying from 1 to 120 nm, as well as consistent size distributions, compositions, and crystallinity with MNPs of different geometrical shapes. Ultrasmall superparamagnetic MNPs have a spherical shape and ultrasmall size, which varies from 4 to 15 nm. Other structures, such as nanocubes [33], nanospheres [34], tetrahedral NPs [35], nanostars [36], nanosheets [37], nanorods [38], nanofibers [39], and nanodots [40], have been described as well.

### 2.1. Physical Methods

When discussing physical methods, different approaches are being used, from “top–down” to “bottom–up”. With the use of high-intensity ball milling, the bulk material is broken down from top down, resulting in various nano-sized particles. However, when using bottom–up approaches, finer nano-sized particles can be produced that have better size distributions. Ball milling is a very straightforward approach that changes the size and texture of particles by finely grinding them with a machine. The material is kept inside a hollow cylindrical pot with steel balls inside. As steel balls and material are in contact with each other, bumping into each other, the material receives kinetic energy from the balls, which causes the material to break down into micro- or nano-sized particles. The size is dependent on the balls-to-particles ratio, as well as the size of the steel balls, its vibration speed, and the time of the grinding. The disadvantage of ball milling is product contamination and an uneven range of sizes compared to particles prepared by chemical methods [41,42]. One of the main advantages of such top–down methods is their relatively low cost since they do not include expensive chemicals and do not generate any harmful waste [41,43,44]. Another advantage is high productivity and its ability to generate and produce materials with less than 20 nm accuracy. On the other hand, particles produced with physical methods do not possess an orderly shapes, which calls for additional processing in order to design desirable nanoparticle shape, which can be achieved with ball milling. A bottom–up approach that makes the MNPs by evaporation from a liquid or gas state is the laser evaporation process, also known as laser ablation. It is a simple way of processing MNPs using high-intensity laser, which is used to evaporate textured materials. In such a process, the vapor of the material is cooled down while being in a gaseous state, which is causing condensation. The result of such processes is the formation of nanoparticles. The main advantage of such an approach is the high precision of the end product, which is of high quality with no specific defects. Furthermore, when compared to chemical methods, the advantage of physical methods is the absence of solvent contamination with the uniformity of nanoparticle distribution. However, physical methods have also been disadvantaged, such as high cost with high energy consumption. More advantages and disadvantages of physical methods are summarized in Table 1. 

### 2.2. Chemical Methods

#### 2.2.1. Co-Precipitation

The co-precipitation synthesis method is the most widely used method, as well as an easy and convenient method for obtaining MNPs, such as Fe_3_O_4_ and γ-Fe_2_O_3_. The utilized metallic precursor determines NP size and shape, as well as its morphological structure and chemical composition. The co-precipitation method involves ferric and ferrous salts in fixed ratios with vigorous mixing under strong basic conditions with a suitable pH value and reaction temperature. As they are dissolved in deionized water, precipitation is followed with ammonium hydroxide base, which is added dropwise. Later, surfactants such as oleic or citric acid are used for stabilization of the colloidal suspension. The equation that describes the chemical process is presented as follows:

Fe^2+^ + 2 Fe^3+^ + 8 OH^-^ → Fe(OH)_2_ + 2 Fe(OH)_3_ → Fe_3_O_4_ + 4 H_2_O

**Figure 3:**
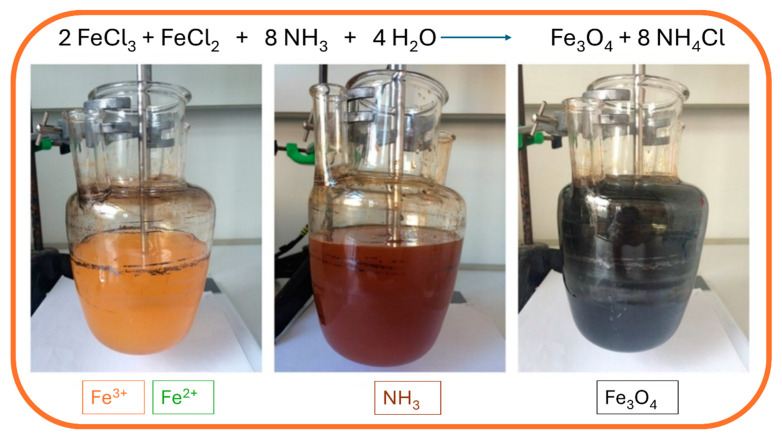
Synthesis of MNPs using the co-precipitation method: mixing of ferrous and ferric solution (yellow; **left**), addition of ammonia hydroxide solution (brown; **middle**), and precipitation of magnetic nanoparticles (black; **right**).

The synthesis is performed under a nitrogen atmosphere, where the nitrogen protects MNPs against critical oxidation but also serves as a factor for reducing the size of the MNPs and contributes to the formation of hydrazine. Hydrazine in the presence of dissolved oxygen results in cationic ammonium hydroxide, which later reacts with ferrous ions (^2+^) and produces ferrite colloid magnetite (Fe_3_O_4_). With that, the yield of MNPs is increased. Synthesis can be performed under different temperatures. When performed under 60 °C or at room temperature, it results in amorphous hydrated oxyhydroxide that can easily be converted to Fe_2_O_3_. However, at higher reaction temperatures of above 80 °C, Fe_3_O_4_ is formed [64]. During the precipitation process, the color of the solution changes from orange to black, which indicates the end of the reaction in the formation of MNPs (Figure 3). 

The black solution is later magnetically decanted and washed several times with deionized water to remove excess chloride ions of the salt. Another washing step can be performed with ethanol to remove any unbound acids used for stabilization. The final step is drying the MNPs to obtain monodispersed MNPs with a narrow size distribution. Bare MNPs can also be coated with different coating materials to prevent agglomeration or to tailor the surface properties of synthesized MNPs. Synthesis of MNPs by co-precipitation method is widely used and promoted in the biomedical field due to its enormous advantages, such as high product yields, crystalline sizes with narrow size distribution of the particles, and environmentally friendly solvents in which the particles are synthesized, as they are normally dissolved in aqueous medium. However, the main disadvantage is the continuous washing and drying, and other limitations that might occur with the co-precipitation method include particle aggregation as a consequence of small particle sizes and shapes, as well as poor dispensability [64]. For example, MNPs were synthesized by the co-precipitation method to investigate their antitumor activity on Ehrlich carcinoma, as reported by Abd-Elghany et al. [65], while Chiarelli et al. developed the synthesis of MNPs by the co-precipitation method for tumor-targeted delivery to intensify the transfer of energy for conventional photon radiotherapy on a cellular basis [66]. Additionally, a study used a similar co-precipitation method of MNP synthesis and utilized both alternating magnetic field and near-infrared laser induction to improve thermal properties in cancer hyperthermia therapy [67]. 

#### 2.2.2. Thermal Decomposition

**Figure 4:**
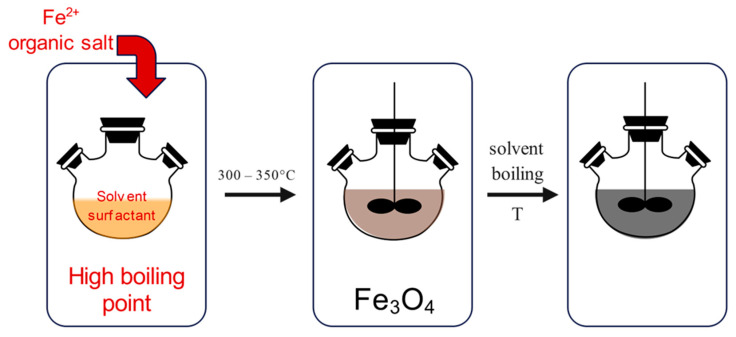
Synthesis of MNPs using the thermal decomposition method: heating of organic iron compound (**left**), followed by thermal decomposition above 300 °C (**middle**), resulting in the synthesis of MNPs (**right**).

The thermal decomposition method (Figure 4) is performed under special conditions, which require high temperatures, ranging from 300 to 350 °C, certain reagents and ligands, as well as an inert atmosphere. The synthesis is performed in a beaker, which contains organic iron compounds, together with oleic acid, which acts as a capping agent. When the reaction is heated to a temperature above 100 °C, it is set for one hour with continuous stirring. After that, the reaction mixture is refluxed for a period of time at certain temperatures.

When the reaction solution is cooled down to room temperature, ethanol is added for the purpose of performing washing steps. If the reaction temperatures are increased, the size of the MNPs can be manipulated since the growth rate increases as well. In addition, increasing the reaction time is causing the increase in the particle sizes and widening their size distributions [68,69]. The thermal decomposition method can provide metal MNPs as well as metal oxide MNPs, depending on the utilized precursors. But to control the particle sizes, expensive and toxic precursors and surfactants must be used, which presents a disadvantage of such methods. For example, MNPs were synthesized by high-temperature thermal decomposition, which was used to conjugate exosomes. Such exosome-conjugated MNPs exhibited excellent size distribution and biocompatibility and were used as promising therapeutic agents treating brain diseases [70]. Furthermore, the continuous flow thermal decomposition method was studied by Glasgow et al. for producing MNPs for biomagnetic imaging and magnetic hyperthermia cancer treatment [27]. Thermal decomposition is an innovative method that produces stabilized monodispersed nanoparticles. This method is also the easiest and most convenient method for producing monodispersed MNPs since it addresses the challenging synthesis of obtaining controlled and monometric sizes of MNPs by optimizing synthesis time and temperature, as well as concentration of all involved reagents (surfactants, stabilizers, reactants, and capping agents).

#### 2.2.3. Microemulsion

**Figure 5:**
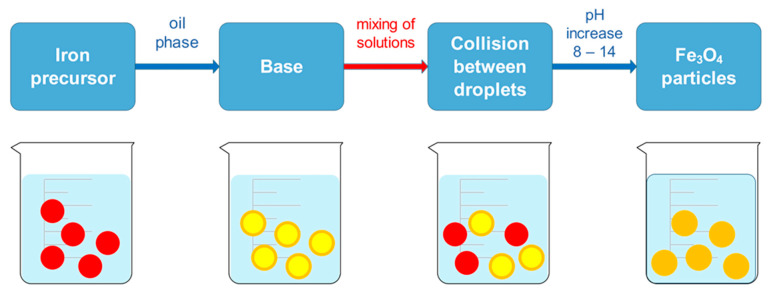
Microemulsion method for synthesizing MNPs.

Simple reaction equipment and the ability to control particle size using different materials are the characteristics that make microemulsion a widely used method to obtain MNPs. The water-in-oil microemulsion method (w/o) uses water droplets as nanoreactors in a continuous organic phase in the presence of surfactant molecules. In this manner, the crystal growth can be better controlled. Using the microemulsion method allows iron precursors to be precipitated as iron oxides in aqueous phase inside micelles. However, the precipitation of iron oxides does not occur in the organic phase, as they are not yet present. As the size of water droplets and used precursors is controlled, the size and morphology of MNPs can also be controlled through utilization of the aforementioned facts. The method is performed using two microemulsions, where the first microemulsion contains metal salts in the aqueous phase, while the second microemulsion contains the precipitating agent, which is added dropwise to the first microemulsion (Figure 5). Continuous stirring at a suitable temperature is performed for a certain period. When the black color is formed, it indicates the formation of MNPs. The precipitate is obtained by centrifugation with several washing steps, using deionized water and ethanol to remove excess surfactant or oil [71,72]. Synthesizing MNPs by microemulsion using NaBH_4_ and FeCl_3_, which resulted in MNPs with good stability and possible application for magnetic hyperthermia cancer therapy, was described by Zhang et al. [73]. 

Compared to traditional methods, synthesis of MNPs based on microemulsion can improve the therapeutic efficacy in drug delivery systems when a typical anti-cancer drug, doxorubicin (Dox), is applied to the synthesis, which demonstrated high drug loading, as reported by Teng [74]. The easy preparation is one of the advantages of the method, as synthesis of MNPs by microemulsion can form at room temperature and can easily be manufactured. In addition, the microemulsion method offers thermodynamic stability and shelf life for the formulation. Such MNPs are nano-sized with minimal aggregation and agglomeration and are of crystalline structure with a high specific surface area. 

#### 2.2.4. Sol–Gel Method

**Figure 6:**
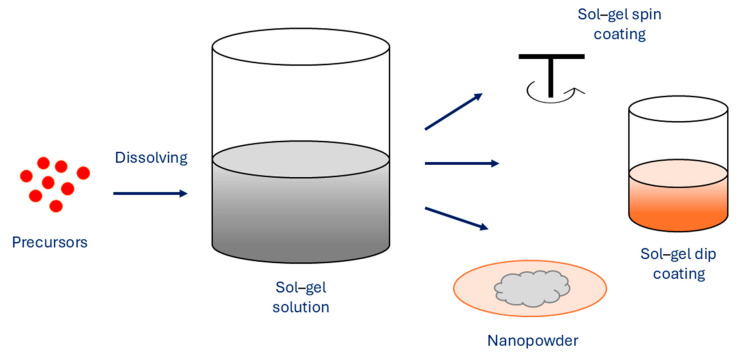
Sol–gel method for synthesis of MNPs.

As one of the essential methods, the sol–gel method uses metal alkoxide precursors or inorganic salts to obtain an oxide skeleton by hydrolysis and polymerization reactions at low temperature in order to synthesize metastable oxides (Figure 6). 

When hydroxylation and condensation of precursors in the aqueous phase occur, the sol is formed. When sol undergoes condensation, it creates a gel, which is made of 3D networks of metal oxides. To remove the solvent, heating is performed in order to obtain MNPs. A surfactant can be added to the reaction solution for system stabilization. The properties of sol–gel synthesized NPs are affected by several factors, such as temperature, pH, concentration of precursors, and nature of the solvent. The sol–gel method exhibits several advantages, which are simple preparation processes that can use cheaper metallic precursors, which result in MNPs with uniform size distribution with high purity, where porosity and its degree can be controlled. Furthermore, the size of the MNPs can be controlled as well, as various sizes can be prepared. The method allows fine control of the chemical composition of MNPs and enables small amounts of dopants to be introduced in the sol. However, the main disadvantages of the sol–gel method are the use of organic solvents and longer reaction times, which consequently result in higher production costs [75,76]. As the use of MNPs to enhance the MRI is dependent on the size of MNPs and their distribution and concentration, the sol–gel method can successfully synthesize suitable MNPs, which act as promising materials for excellent MRI contrast agents, Marashdeh reports [77]. 

#### 2.2.5. Hydrothermal Method

**Figure 7:**
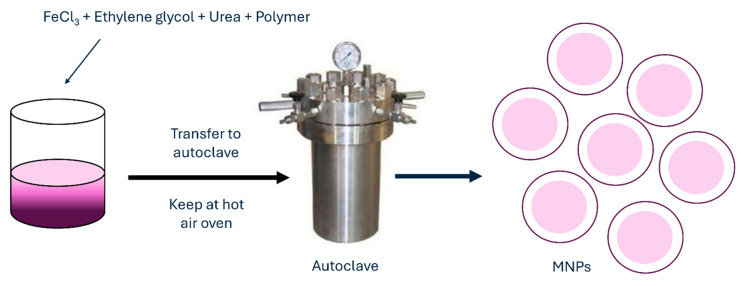
Hydrothermal method for synthesis of MNPs.

Another frequently used method for MNP synthesis is the hydrothermal method, which is also called solvothermal. When using this method, metal precursors in aqueous medium are synthesized without the use of surfactants. They are heated to high reaction temperatures at high reaction pressures in an autoclave (Figure 7). 

An aqueous solution with FeCl_3_ and FeCl_2_ is heated to a desired temperature under argon or any other inert gas when the reducing agent, such as ammonium hydroxide, is added dropwise to the solution under vigorous stirring. When the reaction mixture is kept in an autoclave for a certain period, the product is later washed several times with deionized water to remove any excess ions and salts. Finally, the NPs are dried. Such NPs by hydrothermal method could be synthesized even with the use of surfactants to prevent or reduce aggregation [78] or without them. MNPs synthesized by this method have characteristics of very good and uniform shape and narrow size distribution. Obtained MNPs are highly crystalline crystals with sizes ranging below 100 nm. With the hydrothermal method, nanostructures of different shapes can be achieved, such as hollow nanospheres or nanocubes [79]. With increasing the temperature of the hydrothermal method, the particle size and average crystalline, as well as the coercivity and saturation magnetization of Fe_3_O_4_ NPs, are increasing as well. As reported in research by Köçkar et al., they synthesized MNPs by hydrothermal method for biocompatible docetaxel delivery to breast cancer cells, which improved saturation magnetization, drug loading, release, and cytotoxicity of such MNPs [80]. Another hydrothermal synthesis produced MNPs with significantly narrowed size distribution and improved saturation magnetization, which makes MNPs suitable candidates for magnetic hyperthermia [81]. The hydrothermal synthesis method for the preparation of MNPs offers several advantages, such as easy experimental set-up, low cost, and high yield. In addition, nanomaterials with high vapor pressures can be produced by the hydrothermal method with minimum loss of materials. The compositions of nanomaterials to be synthesized can be well controlled in hydrothermal synthesis through liquid-phase or multiphase chemical reactions. Its disadvantages include the use of expensive equipment and high temperatures. Table 2 summarizes detailed advantages and disadvantages of all chemical methods. 

### 2.3. Biological Methods

Generally, green or biological synthesis of nanomaterials utilizes the synthesis of various nanomaterials, such as MNPs, without including hazardous materials that result in toxic by-products. Furthermore, the green method of synthesis follows the principles of environmentally friendly approaches for the synthesis of MNPs, where it does not pose any harmful risks for the surrounding environment or human health. It is true that traditional synthesis methods and approaches can produce larger amounts of MNPs with expected morphology profiles and sizes. However, such methods and approaches require immense and complex construction, which is also cost-related. In contrast to traditional chemical and physical synthesis methods, green biological synthesis offers numerous advantages, such as lower toxicity, simplicity, cost-effectiveness, and creating fewer wastes [110]. Biological or green synthesis is a bottom–up approach, where metal atoms are assembled and formed into clusters, which eventually become MNPs. For stabilization of MNPs during the synthesis process, biological compounds that are present in green materials act both as capping and reducing agents. Therefore, the size and shape of MNPs can be controlled and used in various bioapplications. 

**Figure 8:**
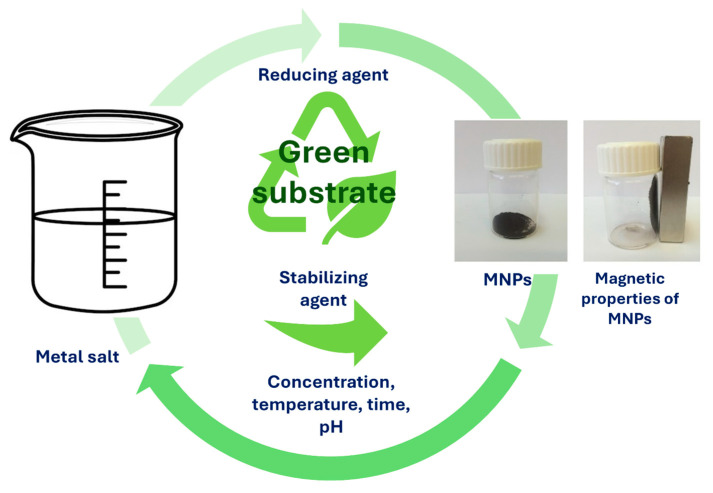
Simple synthesis process of MNPs using green substrates.

Figure 8 illustrates the simple MNP synthesis, where the required materials as precursors are metal salts in the presence of only green substrate. During MNP synthesis, different parameters can be adjusted, such as reaction time, reaction temperature, pH of the solution, metal salt concentration, and green substrate concentration, in order to achieve the improved properties that are needed for desired bio-applications [12,111,112]. Green synthesis of MNPs, where natural materials, such as plants and microorganisms (e.g., bacteria), fungi, algae, or yeasts, are utilized, is a remarkable process to produce MNPs based on iron oxides. However, phytochemicals that are present in plant extracts have an incredibly high attitude for the reduction in metal ions during shorter times, when compared to bacteria, fungi, algae, or yeasts. Therefore, longer incubation times are required. In green synthesis, the use of plant extract is more desirable over microorganisms, as they require more complicated culturing. Moreover, the compounds present in plant extracts, such as sugars, terpenoids, and proteins, help with the bioreduction of metal ions. Synthesizing MNPs with green-oriented synthesis is expanding the range of MNP applications [113]. Nevertheless, even plant parts as substrates, such as seeds, fruits, peels, or leaves, were used as capping or reducing agents for the production and synthesis of MNPs. As plant extracts possess valuable phytochemicals, such as glycosides, flavonoids, and polyphenols, they too can act as stabilizing, reducing, or capping agents. As reported by Rizvi et al., MNPs were synthesized by the green method using a water extract of pitaya, which can be easily separated from the solution and may find possible applications in targeted drug delivery systems, diagnostics, biosensors, and many others [113]. Pillai et al. biosynthesized MNPs using *Pimenta dioica* leaf extract, which reduced the cell viability and had an efficient inhibitory effect on human colon cancer cells [114]. The advantages and disadvantages of biological methods are presented in Table 3. 

## 3. Strategies in MNP Functionalization

There are several established strategies for stabilizing MNPs in cellular environments. However, these methods often involve attaching ligands to the NP surface, which can compromise targeting specificity. This is because the attached stabilizing ligands can significantly increase the MNPs’ hydrodynamic diameter, altering key properties like circulation time in the bloodstream, cellular uptake, and mobility. A smaller hydrodynamic radius is generally preferred for efficient transmembrane passage and excretion. Thus, when performing surface functionalization, it is crucial to carefully consider both the size of the MNPs and the selection of an appropriate ligand to ensure favorable bio-distribution. Another important consideration is the limited commercial availability of some stabilizing agents. Maintaining colloidal stability while integrating ligands onto MNPs during functionalization is a significant challenge. The process is highly dependent on the nanoparticle’s composition, leading to the development of various methods tailored to different surface chemistries. 

**Figure 9:**
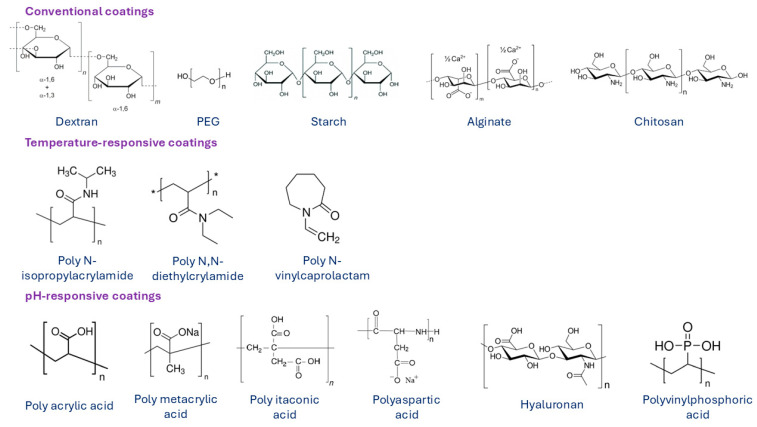
Various surface coatings of MNPs based on natural and synthetic polymers. Summarized from [147].

In order to improve the biocompatibility, biodegradability, and non-toxicity of MNPs, their surface must be modified with biocompatible coating materials. The modification is performed either during or after preparation and is applied to the synthesis process to stabilize the MNPs in biological medium. Surface modification of MNPs also prevents possible oxidation and enables attachment of functional groups, such as targeting ligands, drugs, or other biologically active compounds. Materials that are used to coat MNPs are typically polymers, fatty acids (such as oleic [123], citric [124], stearic [125], lauric [126] or other acids), or amino acids (such as cysteine [127], lysine [128], tyrosine [129], arginine [130], phenyl alanine [131]), metals (such as gold [132], silver [133] or gadolinium), and oxides (such as silica [71] and titanium [134]). Among all suitable materials, organic and inorganic polymers are the most widely used and desirable coating materials. Over the last decade, various natural and synthetic polymers were applied in the synthesis process of MNPs, such as chitosan [135], dextran [136], alginate [137], gelatin [138], starch [139], albumin [140], casein [141], as well as polyethylene glycol (PEG) [142], polyvinyl pyrrolidone (PVP) [143], polyvinyl alcohol (PVA) [143], polydopamine [144], polylactic-co-glycolic acid (PLGA) [145], and dendrimers [146]. Some examples of polymeric coatings are presented in Figure 9. Conventional polymers are used as coating materials for the synthesis of most commercial MNP formulations. However, for some applications, MNPs are surface-functionalized with temperature- or pH-responsive polymer coating materials. 

Broadly, three main classes of MNPs are recognized based on their surface modification: (i) noble plasmonic metals like gold (Au) and silver (Ag), which are typically functionalized with organic groups such as thiols (-SH), amines (-NH_2_), and cyanides (-CN); (ii) oxides, such as iron oxides, which can be modified with hydroxyl (-OH) and acidic groups through oxygen bonds; and (iii) binary compounds, including fluorescent semiconductor MNPs, which have a higher affinity for groups like -OH, -SH, and -NH_2_.

### 3.1. Surface Functionalization of MNPs

Surface functionalization of MNPs is a powerful technique to address issues related to MNP toxicity and cellular absorption while also enhancing properties beneficial for biomedical applications. Initial functionalization steps expose various functional groups on the MNPs surface, giving them specific chemical properties. Basic surface modification involves adding homo- or hetero-bifunctional groups, such as -NH_2_, carboxyl (-COOH), -OH, or -SH, to the MNPs surface. These functional groups create bonds with the NPs surface, facilitating the attachment of biomolecules or other entities. Surface modification is essential to stabilize nanoparticles by preventing agglomeration or oxidation and to improve their properties for biomedical use. Different organic groups form bonds with NP surfaces, preventing agglomeration and enhancing interactions with biological targets. These enhanced interactions allow for more precise targeting or sensing of specific molecules.

To integrate various inorganic and organic molecules onto the MNPs surface, both covalent and noncovalent forces, such as electrostatic forces, hydrogen bonds, and van der Waals forces, are employed in surface functionalization. Covalent bonds between MNP surfaces and ligands typically involve multiple linker molecules, while noncovalent interactions are simpler and preserve the structure and function of the molecules involved. Factors like ionic strength and pH can easily manipulate noncovalent interactions. Different nanostructures incorporate distinct functional groups during early surface functionalization steps, generally involving the use of homo- or hetero-bifunctional cross-linkers to add organic functional groups (-NH_2_, -COOH, -OH, and -SH) that bind biomolecules. Key aspects of surface functionalization include (i) maintaining MNP stability against oxidation and agglomeration, (ii) ensuring compatibility with subsequent phases, such as water solubility when appropriate ligands are attached, and (iii) aiding in the self-organization of MNPs. This surface modification minimizes compatibility issues between different phases in the resulting nanocomposite [148,149,150].

### 3.2. Polymer-Functionalized MNPs

The most commonly used polymers as coating materials for surface modification of MNPs are dextran and PEG, as they are in general regarded as safe (GRAS) by the United States Food and Drug Administration (FDA). Another reason for its extensive use, especially for biomedical application, is the fact that dextran and PEG are not rapidly recognizable by liver or spleen macrophages when they are intravenously administered [151,152,153]. PEG and materials that are PEGylated (such as starch) possess incredible biocompatibility and are often prolonging vascular circulation on MNPs. Furthermore, the surface density and molecular weight of PEG coatings are the most important parameters, which influence the cytotoxicity, dispersion stability, and blood circulation time of MNPs [154]. As reported by Harris, PEGylated MNPs were investigated as drug delivery vehicles, where anticancer drugs interacted with DNA to explore their use and potential to change drugs’ chemistry before delivery [155]. In addition, PEGylated MNPs also show enhanced surface functionality while applying drug daunorubicin, which demonstrated excellent drug loading and stability, a report by Mohanta et al. says [156]. PEGylated MNPs were also investigated for oxidative stress in cultures of ovarian tissue, according to research by Karimi et al. [157]. In a similar manner, polymer dextran and its derivatives, such as carboxymethyl dextran [158,159,160] and carboxydextran [161], are also known for their incredible biocompatibility. However, although dextran has no direct cytotoxic effect, its dextran shell degradation may have some influence on cellular processes [162]. A study by Das et al. developed a simple synthesis method to develop carboxymethyl dextran-coated MNPs, which are suitable for MRI applications [163]. Besides the standard polymers mentioned, more research has been focused on the development of stimuli-responsive coatings based on polymers. The main characteristic of stimuli-responsive polymers is their alteration of swelling behavior, where they respond to the changes in environment, such as pH, temperature, ionic strength, magnetic field, and light. The pH- and temperature-responsive polymers have been used and employed the most commonly [156,164]. When applying pH-responsive polymers, their shape and volume change with the change in the pH. Due to altered protonation of carboxyl groups, polymers with weakly acidic pendant groups exhibit pH responsiveness. When applying temperature-responsive polymers, their chemical and physical properties change with the change in the surrounding temperature. They can undergo a phase transition when the temperature is increased above the lower critical solution temperature [165,166,167]. MNPs can also be entrapped inside various vesicles, such as liposomes [168,169,170] or different polymeric micelles [171]. Encapsulation of MNPs is different from the surface functionalization methods; however, encapsulation provides similar characteristics and advantages, where their biocompatibility is enhanced and the in vivo performance for the attachment of functional groups is improved. Liposomes are amphiphilic phospholipids, which are assembled in the bilayer of their hollow sphere structure. Hydrophobic and hydrophilic MNPs are usually entrapped in its liposome bilayer and in its inner core, respectively. Magnetoliposomes are liposomes that are used for entrapment of MNPs [172,173,174]. A polymeric micelle is a self-assembled NP made from an amphiphilic block copolymer in which a hydrophobic compartment entraps hydrophobic superparamagnetic iron MNPs (SPION), while a hydrophilic shell stabilizes the MNPs in solutions or biological medium [171].

## 4. Magnetic Properties of MNPs

MNPs exhibit unique magnetic properties such as saturation magnetization, remanence, and coercivity, which are pivotal in determining their functionality in biomedical targeting mechanisms. Saturation magnetization refers to the maximum magnetization that MNPs can achieve under an external magnetic field, directly influencing their ability to be manipulated or concentrated at target sites. Saturation magnetization is crucial because it determines the strength of the magnetic response of MNPs under an external magnetic field. Higher saturation magnetization allows for stronger magnetic forces to be applied, improving the efficiency of targeting and concentrating the nanoparticles at a specific site within the body. Remanence, the residual magnetization after the external field is removed, plays a crucial role in maintaining the MNPs’ alignment and interaction with biological targets, particularly in applications like magnetic hyperthermia and drug delivery. High remanence and coercivity, the resistance to demagnetization, can lead to the formation of particle clusters, which may impact the MNPs’ dispersion and targeting efficiency. However, for applications like targeted drug delivery, lower remanence and coercivity are often desirable to prevent unwanted agglomeration and ensure that the particles do not remain magnetized, which could lead to off-target effects. The balance between these parameters is critical; for instance, lower coercivity and remanence are preferred for superparamagnetic nanoparticles, which exhibit no residual magnetization in the absence of a magnetic field, allowing for better control and reduced side effects in vivo. Recent advancements emphasize optimizing these magnetic parameters to enhance the specificity and efficacy of MNPs in targeted therapies, ensuring precise delivery while minimizing off-target effects [175,176].

Recent studies have highlighted the significant impact of external magnetic fields on the delivery efficiency of magnetic nanoparticles (MNPs) to tumor sites. The use of a magnetic field can dramatically enhance the concentration of MNPs at the targeted site compared to passive diffusion alone. For instance, one study demonstrated that applying a magnetic field can increase the delivery of MNPs to tumors by up to five times compared to non-magnetically guided methods, particularly in gliomas where precision is critical [177]. The effectiveness of magnetic targeting depends on several factors, including the size of the nanoparticles and the strength and positioning of the magnetic field. Smaller nanoparticles, such as ultrasmall superparamagnetic iron oxide nanoparticles (USPIOs), are particularly promising because they can penetrate deeper into the tumor tissue under the influence of a magnetic field, thereby increasing the therapeutic impact [178]. Additionally, the simulation of magnetic nanoparticle delivery shows that when a magnetic field is used, a high percentage (over 90%) of nanoparticles can be directed to the tumor site depending on the distance from the magnet and the size of the nanoparticles used [179]. The study by Camargo Casallas et al. provides a comprehensive study on the use of finite element analysis (FEA) to evaluate and optimize the magnetic targeting efficiency of MNPs in biomedical applications. The targeting efficiency of MNPs with various sizes (from 50 nm to 200 nm) was modeled under a constant magnetic field of 0.12 T using a computational tool based on the FEA. Their results showed that MNPs targeting efficiency was the highest with simulated magnetic fields located 5 cm, 7.5 cm, and 15 cm away from the tumor when using MNPs with the sizes of 50 nm and 100 nm [179].

The balance between the amount of MNPs needed for effective targeting using an external magnetic field and the maximum dose that can be safely administered in vivo without causing toxicity is crucial for the success of nanoparticle-based therapies. The amount of MNPs required for effective targeting typically depends on factors such as the strength of the external magnetic field, the magnetic properties of the nanoparticles (like magnetic moment), and the distance between the magnetic source and the target tissue. For instance, research has demonstrated that doses around 100 mg/kg of human body led to apoptosis of circulating erythrocytes in vivo [180] and can be effectively guided to tumor sites under the influence of an external magnetic field [181]. 

A study by Tian et al. aimed to develop a novel cable-transmission magnetically controlled capsule endoscopy system for detecting gastrointestinal diseases and assessing its safety and feasibility through clinical trials [182]. Another study by Liu et al. reports on applying low-frequency magnetic field in the treatment of glioblastoma, where they specifically emphasize current understanding of the mechanisms by which low-frequency magnetic field can mediate anticancer effects [183].

## 5. MNPS in Biomedical Applications

MNPs have been the focus of many medical-related applications due to their unique attributes, such as super magnetic properties, size and shape, a larger surface-to-volume ratio, but most importantly, biocompatibility, which makes MNPs a desirable candidate for various biomedical applications. MNPs have the potential to alter the magnetic field surrounding them since the external magnetic field at dipoles can produce different types of force and torque, which causes the energy to be rotated or radiated away from the source of magnetic field. In addition to extensive drug delivery systems with gene delivery and administration, MNPs are also applied to cell separation, biomarker development, biomedical imaging, bacterial detection, and hyperthermia. As MNPs are composed of a wide range of materials with various physical and magnetic properties that vary depending on their application, the most important feature is the high biocompatibility and low toxicity of synthesized nanomaterials. 

### 5.1. MNPs in Drug Delivery

Drug delivery defines a process of delivering a specified dosage of a bioactive compound or therapeutic, such as proteins, genes, naturally occurring or artificial medicines, to a specified organ or tissue in the human body. Drug carriers can increase therapeutic efficiency and protect the compound or therapeutic agent while transporting hydrophilic and lipophilic drugs by avoiding the breakdown caused by enzymes [184]. Different nano-delivery systems improve various administrative routes of drug biodistribution with minimized side effects since nanocarriers deliver pharmaceuticals or drugs to the desired targeted site either passively or actively [185]. For a reliable drug delivery system, the blood circulation system must offer minimum dosage loss and minimum function loss. Furthermore, the drug or therapeutic agent must affect only the tissue or organ to which the drug is targeted and not cause any damage to other healthy cells [186,187]. As MNPs are being explored, their inherent properties allow them to be incredible tools in medical theranostics. The most important and leading biomedical applications are in relation to therapeutics and diagnostics for treating cancer. By that, meaning mostly to treat cancer types that cannot be treated surgically. Profound and focused research is investigating the understanding of cancer and its progressiveness by ways of developing new approaches to end it. In an effort to end, treat, and stop the cancer process, innovative methods and therapeutic approaches are being developed using various chemotherapies and radiotherapies. A variety of cancer therapies are being developed extensively, such as targeted therapy, magnetic drug delivery, magnetic fluid hyperthermia, immunotherapy, hormonal therapy, and angiogenesis inhibitors. Many studies investigate the use of MNPs for magnetic hyperthermia since MNPs have high magnetic heating efficiency, as reported by Zuo et al. [188]. Encapsulated MNPs are an excellent tool for hyperthermia therapy since they display biocompatible properties based on cell viability. Such MNPs increase cellular uptake in the presence of conjugated biotin and exhibit effective killing activities of cancer cells, Nguyen et al. report [189]. Therefore, the main aim of applying MNPs in drug delivery systems is to continuously be searching for alternatives to treat cancer and consequently to avoid or prevent undesired side effects and cytotoxic medication utilization when applied through injection into the blood circulation system. MNPs that are based on different ferrofluids and at the same time carry an anticancer drug are administered intravenously in order to localize at the desired location with the use of an external magnetic field. Magnetic targeted treatment is based on the use of magnetic field and its variation to produce magnetic force on MNPs. 

**Figure 10:**
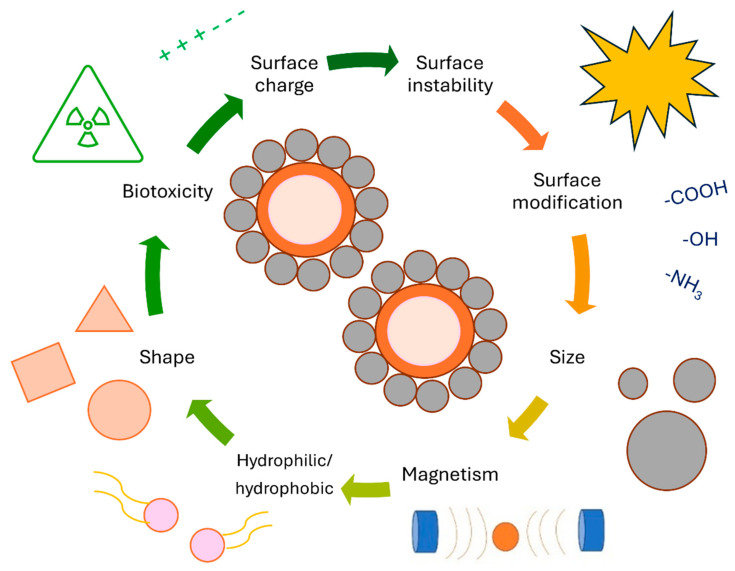
MNPs with their physicochemical limitations related to drug delivery systems.

MRI efficiency of magnetic drug delivery systems is much higher than that of polymer, lipid membrane, and micelle-based drug delivery systems, potentially allowing for in vivo monitoring of drug distribution [131,190,191]. Moreover, in vitro MRI analysis revealed the noticeable potential ability of synthesized MNPs to generate negative contrast by reducing MR signal intensities, which could make the MNPs a promising tool for Alzheimer’s theranostic application, a report by Chen says [192]. Since MNPs are biodegradable and biocompatible, they are ideal tools for various biomedical applications. Of course, some physiochemical limitations, shown in Figure 10, may occur. MNPs are frequently ineffective as drug nanocarriers due to their rapid adaptation and delayed plasma discharge by the reticuloendothelial system (RES) macrophage tissues before particulate particles reach the tissues or cells of concern [193]. Even though researchers have developed numerous techniques and approaches to load MNPs with drugs, these nanocarriers may perform awfully when significant drug release is necessary at the target site. Despite their larger surface area, these MNPs have weak magnetic properties, making them less suitable for drug loading [194]. Drugs could be directly attached to MNPs through covalent binding, adsorption, electrostatic attraction, and encapsulation. Depending on their size and surface chemistry, drugs or MNP complexes can be delivered to affected tissues either actively or passively. Increasing capillary permeability and possession in tumor tissues results in passive targeting, while in active targeting, MNPs are taken by tumor tissues through the recognition of targeting agents on the MNPs surface [195]. A magnetic field is used to attract particles to the affected zone after adhering recognized ligands (such as antigens) to the outside of MNPs [196]. Surface properties, size, shape, and magnetic properties, such as coercivity, magnetic moment, and remanence, influence the performance of MNPs. Immunological components such as RESs are necessary for minimizing the activity of RES and increasing the half-life of RES in the circulatory system. By covering MNPs with neutral or hydrophilic materials, such as polymers and biomolecules, the half-life of the systemic circulation has been prolonged from minutes to hours or even days. However, despite all attempts, it remains doubtful whether RES can be completely removed. Another option would be to minimize the size of the MNPs. There may also be toxicological consequences associated with the unwanted movement to other parts of the body [70,110]. Several diseases beside cancer, such as anemia, chronic kidney disease, and musculoskeletal disorders, can be treated with MNPs [197]. By acquiring low dosages and side effects, MNPs attain efficient strategic ratios in these applications. Therefore, superparamagnetic MNPs with external magnetic fields appear as a promising alternative for drug delivery to inflammatory areas [197,198]. 

**Figure 11:**
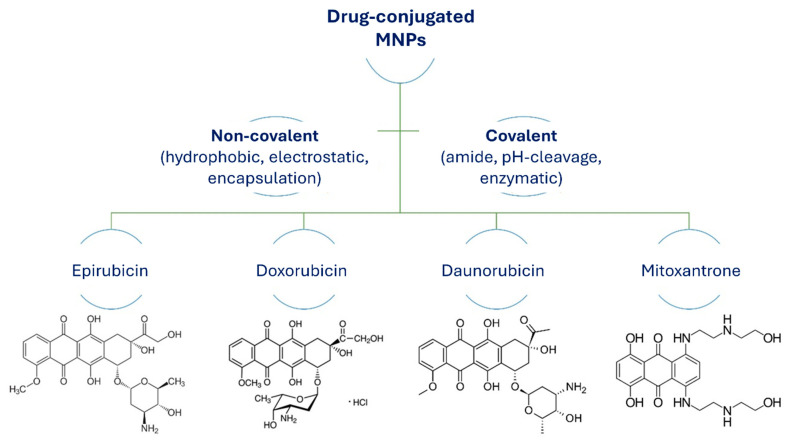
Structures of anticancer drugs conjugated to MNPs via covalent or noncovalent pathways.

The most common pathways to conjugate drugs with MNPs for drug delivery applications and hyperthermia are chemical and physical functionalization. These methods to attach drugs to MNPs include noncovalent conjugation through direct physical adsorption, dipole–dipole interactions (by hydrogen binding), electrostatic interactions, hydrophobic interactions, and encapsulation. Covalent chemical reactions between drug molecules and the surface functionalized MNPs are through active functional groups (Figure 11). 

In a drug delivery system, a drug or a therapeutic compound is conjugated (adsorbed, attached, or encapsulated) to a magnetic nanocarrier while later being administered and released. Generally, there are two main routes for drug release: locally and externally activated. Locally activated drug release takes place by simple diffusion or through various endocytic mechanisms that require either chemical or biochemical stimuli, for example, pH, enzymatic activities, hydrolysis, and others. By that stimulus, the drug release is triggered. For polymeric nanostructures or for polymer-modified MNPs, the endocytic, phagocytic, or receptor-mediated pathway for cellular internalization is determined by their surface charge and size. However, externally activated targeting is dependent on other external factors, such as temperature, magnets, ultrasound, and light. Such nanocarriers, which are stimuli-responsive, have been proven to be improved for in vivo and in vitro drug releases [197]. 

### 5.2. Key Factors Affecting Drug Delivery Efficiency

To excel in drug delivery, MNPs must possess four specific qualities, such as (1) uniformity in size and shape (monodispersity), (2) magnetic responsiveness (superparamagnetism), (3) stability, and (4) compatibility with living organisms (biocompatibility). Various methods have successfully synthesized monodispersed nanoparticles like iron (Fe), cobalt (Co), γ-Fe_2_O_3_, and Fe_3_O_4_. Consequently, SPIONs have gained approval from the Food and Drug Administration (FDA) as contrast agents for MRI scans [199,200]. FDA approval assures the complete safety of iron oxides, particularly magnetite, which is intended for human use. Superparamagnetism refers to the magnetic behavior of particles, which depends on the presence or absence of an external magnetic field [201,202,203]. These properties remain stable even after removing the external field. Superparamagnetic properties are crucial during drug delivery as they prevent agglomerations that could potentially cause blood vessel blockage (embolism). The threshold size for exhibiting superparamagnetism varies depending on the material. For example, ferromagnetic nanoparticles with a size smaller than 20 nm generally have a single magnetic domain with a collective magnetization direction. For iron-based nanoparticles, they become superparamagnetic at sizes smaller than 25 nm, while pure iron nanoparticles exhibit superparamagnetism at sizes smaller than 3 nm [204,205,206,207]. Particle stability is another requirement for biomedical applications of MNPs. This can be achieved by using core–shell MNPs, where a metal or metallic oxide core is enclosed within an inorganic or polymeric coating. This coating ensures the particles remain permanently biocompatible and can also serve as a support for biomolecules [208]. MNPs intended for biological applications need to be small, with a hydrodynamic size less than 50 nm. This size allows them to easily pass through blood vessel walls and avoids uptake by the reticuloendothelial system (RES)—a part of the immune system that includes phagocyte cells and is present in liver, adipose tissue, bone marrow, spleen, and basement membrane [209,210,211,212,213]. As previously discussed, the combination of a desired drug and a magnetically active component is often formulated into a stable system for controlled release within the bloodstream. The rate at which the drug is released can be precisely controlled by manipulating the magnetic field. This targeted therapy approach offers several advantages, including increased accuracy, precise targeting, and a high capacity to deliver drugs, which can help minimize toxic side effects and enhance therapeutic efficacy [214]. The successful utilization of MNPs for biomedical applications such as targeted drug delivery relies on several factors related to the size and magnetic properties of the biocompatible nanoparticles. Parameters like the physical and chemical characteristics of the drug-loaded MNPs, the strength and geometry of the magnetic field, the depth of the target tissue, the rate of blood flow, and the vascular supply all contribute to determining the effectiveness of this drug delivery method. Increasing the magnetization of the nanoparticles can be beneficial for facilitating their manipulation within drug delivery systems. Additionally, the nanoparticles need to be small enough to exhibit superparamagnetism, which prevents them from clumping together once the magnetic field is turned off and allows them to remain in circulation without being filtered out by the body's natural systems, such as the liver or immune system. Superparamagnetic nanosystems are preferred because they can be magnetized when exposed to a magnetic field but do not retain any permanent magnetization (remanence) once the field is removed. Superparamagnetism is a result of thermal effects within the material, where thermal fluctuations are strong enough to spontaneously demagnetize previously magnetized particles. As a result, these particles have zero coercivity and do not exhibit hysteresis [215]. 

### 5.3. Mechanism of Drug Delivery

**Figure 12:**
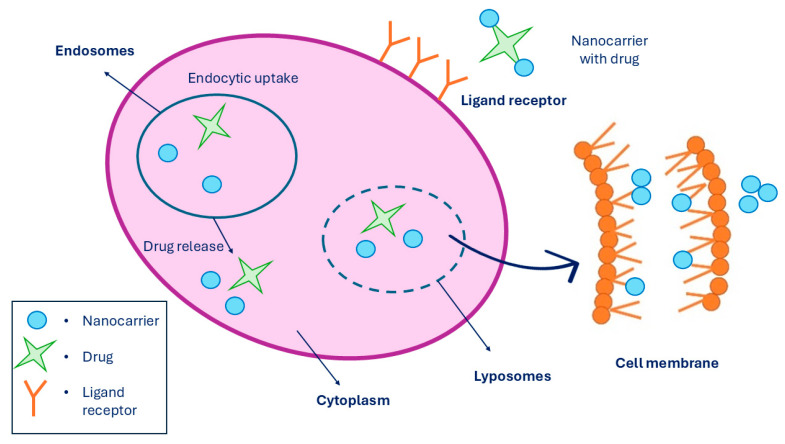
Targeted drug release system performed in a cell membrane, where it shows the linkage of MNPs to the receptors while being entered into the cell via endocytosis, followed by drug release. Summarized from [218].

A targeted drug delivery system is a system where the pharmaceutical drug is being transported to the desired location inside the body with the purpose to prevent any unnecessary interactions with other healthy tissues and therefore to avoid side effects. Since non-targeted drug delivery can lead to some undesirable side effects on healthy cells, targeted drug delivery improves the effect of the drug being administered and can reduce the drug dosage to a minimum. However, targeted drug release is a process performed in three steps. Firstly, the nanocarrier binds the receptor through multivalent receptor ligand interactions of the targeted cell. Secondly, drug-loaded nanocarriers enter the cell through endocytosis. And lastly, when the drug-loaded nanocarrier is inside the cell, it releases the drug at the location. Moreover, targeted drug delivery can be performed in the cytosol and in the cell membrane by lipid membrane interaction [216], which is presented in Figure 12. The last step, which is the drug release, can be performed in two ways: cleavage of the linker and carrier control. Linkers can be of two types, such as cleavable and non-cleavable. They are known as a part of fusion proteins that separate multiple domains in a single protein. While cleavable linkers are breaking bonds under suitable conditions, such as when the pH of the environment promotes the cleavage, non-cleavable linkers do not perform cleavage triggered by any chemical reagents [217]. There are various mechanisms for drug release through linker cleavage, such as amide, ester, hydrazone, and disulfide hydrolysis, as well as hypoxia activation, photochemistry, and thermolysis. While ester linkers act under basic pH conditions, amide linkers are used to formulate the drug with the nanocarrier. When chemical hydrolysis of the amide bond is established, elevated temperatures with a strong base or acid are required.

Although the hydrazone bond is stable at pH 7.4, the optimal pH for the cleavage to take place is around pH 5. When an electrochemical reaction in the cytoplasm is initiated, the cleavage of the disulfide bond takes place, which is triggered by the thiol group. When the oxygen supply is low, the hypoxic condition occurs inside cell microenvironments [219]. Photocleavable linker is involved in photochemistry, where it is initiated by light. When thermal stimulus is present in the microenvironment, thermolysis is performed, which promotes the cleavage of chemical bonds with the drug linker [218,220]. For drug loading and release, noncovalent mechanisms can be used. They are performed by encapsulation or other interactions with the carrier, where its particle shape and size have an important role in regulating the biology of the mechanism. When encapsulation is being used for drug delivery, the drug is loaded into the suitable nanocarrier, where it is later released through controlled diffusion [221,222]. Noncovalent conjugation in drug delivery is employed in many mechanisms due to its simple and effective approach. These mechanisms are exploited by many natural biomacromolecules with the use of electrostatic and dipole–dipole interactions, such as hydrogen binding, van der Waals forces (hydrophobic), encapsulation, and coordination, in order to create self-assembled structures. Based on the investigations and research already conducted, it is evident that many new emerging pharmaceuticals will be based on noncovalent interactions between many building blocks and their responsive behavior to selected nanocarriers. The FDA already approved some formulations, which have drugs noncovalently entrapped, such as Doxil®, DaunoXome®, Myocet®, and others. Another example are doxorubicin (Dox)-loaded polymeric micelles, which were found therapeutically active when they were noncovalently entrapped into their micellar structure instead of when they were covalently bound through amide bonds [223]. The main challenge and the key to every investigation is to find optimal interactions between the drug and the nanocarrier to avoid uncontrolled release and at the same time enable time-controlled responsive delivery. The main advantage of noncovalent conjugation is the lack of covalent modification that would affect their structure and the body it is being released into. It is also a simple procedure with no need for aggressive chemical reagents, and by that, reducing the toxic effect by creating an effective tool for potential clinical applications. Absorption of the drug is the movement of the selected drug through the circulation system of the body and can occur in five different routes, such as transport protein-mediated absorption, transcellular absorption, paracellular absorption, pinocytosis, and endocytosis. The main obstacles to drug absorption are low aqueous solubility and permeability. On the other hand, adsorption of a drug-loaded nanocarrier occurs through interactions with organisms or through biobarrier permeability. Using different nanostructured carriers can help to overcome any drawbacks to avoid loss of effectiveness and to improve the efficiency of the active nanocarrier-loaded drug [224]. Dox is the most commonly studied and used chemotherapeutic anticancer drug, together with its related anthracycline family of epirubicin and daunorubicin. They are being used in clinical applications for treating a wide range of cancers and tumors, which include leukemia, lymphoma, lung, ovarian, and breast cancer. Dox-loaded MNPs were studied by Unnikrishnan et al. for investigating their loading and controlled release, as well as demonstrating a decrease in cytotoxicity in folate receptor-positive cells [225], while Mohapatra et al. synthesized MNPs, which were carboxymethyl stabilized, to evaluate in vitro anti-tumor potential by loading the anticancer drug Dox [226]. However, the side effects of these chemotherapeutic drugs limit their dosage and administration. For example, Dox can cause heart failure and myocardial damage if the dosage exceeds the total cumulative dosage. Therefore, an urge to develop effective, safe, and more selective delivery systems for such drugs is in place. Some MNP-based nanocarriers used for drug delivery of Dox via a noncovalent approach are presented in Table 4. 

Encapsulation of nanocarriers, also known as nanoencapsulation, is a way of enhancing magnetic delivery systems where the MNPs are coated with porous materials, usually silica. Nanoencapsulation of chemotherapeutic drugs increases their targeting ability, specificity, and efficacy. In addition, nanoencapsulation of MNPs can prevent any enzymatic or biologic degradation of the drug, which results in the fact that lower concentrations can be sufficient for achieving the desired chemotherapeutic effect. It also enhances bioavailability and increases cellular uptake. While the size of MNPs and their distribution are important to determine cellular uptake and penetration that occurs through biobarriers, their surface chemistry is important to determine the in vivo performance. Different release mechanisms are highly dependent on the nature of a chemotherapeutic drug and the type of MNPs that are used for certain targeted delivery systems. Singh et al. addressed the problems of undesired surface oxidation and agglomeration of MNPs by developing casein-coated MNPs for cytarabine encapsulation and evaluated their anticancer activities and in vitro drug release [233]. To allow controlled or environmentally responsive drug release, the covalent bond should be biocompatible and intracellularly biodegradable or cleavable on demand. In general, MNPs in which drugs are covalently conjugated to the surface of the MNPs exhibit poor drug entrapment efficiency and have difficulty releasing drugs when compared to noncovalent counterconjugates. Furthermore, when drugs are covalently linked to the surface of MNPs, residual catalyst concentrations may result in in vivo toxicity. However, it is important that the structure and orientation of the drug do not alter during performed covalent attachment since it may cause a change in its biological activity. Binding structures that occur are commonly enzymatically cleavable bonds or amide linkages, which are pH degradable. Some examples of covalent attachment of drug Dox onto different MNPs are summarized in Table 5. 

Bonds commonly used to covalently conjugate different drugs to MNPs are amide bonds, which are generally susceptible to hydrolysis at lower pH values. They are also resistant to protease degradation, which occurs inside the cell. Nevertheless, amide bonds are very sturdy and can enhance the thermal stability of some chemotherapeutics in order to prolong their lifetime in the bloodstream.

### 5.4. Toxicity and Limitations of MNPs in Drug Delivery Systems

Nanotoxicology represents a vast field of research, yet further investigations are imperative to enhance our comprehension of the human body's reactions to nanoparticles [238]. The size of these particles is a pivotal factor influencing their toxicity, presenting challenges for nanomedicine, primarily in terms of the environmental repercussions of nanoscale materials and the potential toxicity of novel pharmaceutical and biomedical products. When delving into nanoparticle toxicity, generalization becomes intricate due to the multitude of factors at play, including chemical composition, dose, size, administration method, solubility, biodegradability, biodistribution, surface chemistry, pharmacokinetics, shape, and structure. Evaluating the risks and benefits is essential, as opposed to other biomedical breakthroughs, to assess whether the potential risks are justifiable. Notably, toxicity associated with MNPs often arises from serum proteins binding to their surface, altering the cellular medium composition. Key determinants of cytotoxicity include surface area, shape, size, composition, and coatings of nanoparticles, with modifications to the nanoparticle surface serving as a crucial strategy to minimize toxic effects [239]. Coated nanoparticles exhibit lower toxicity due to the presence of a biocompatible coating, which limits the sites available for protein and ion adsorption. Surface coverage, a vital parameter, influences cellular uptake, impacting opsonization, endocytosis, and plasma half-life. The internalization mechanisms and biodistribution of MNPs are intricately linked to surface chemistry and hydrodynamic sizes. Opsonization by plasma proteins leads to macrophage-mediated removal, with liver and spleen exhibiting the highest overall uptake. Long-chain polymer-coated MNPs are less cell-toxic compared to their shorter-chain counterparts. Magnetic drug delivery faces limitations related to external field strength, nanoparticle size, and residence time control in the bloodstream. Targeting efficiency depends on blood velocity and proximity of the magnetic field source. While nanoparticle sizes facilitate diffusive permeability in capillaries, the main limitation lies in their residence time in the bloodstream. Passive targeting using conventional nanoparticles is restricted to mononuclear phagocyte system organs, necessitating active targeting strategies for other tumor tissues due to short circulation times and low nanoparticle concentrations despite the enhanced permeability and retention effect [181,240,241].

## 6. Current Challenges and Future Perspectives

Despite the great potential MNPs as biomaterials are displaying in many applications, there are still questions over their overall usage, especially in in vivo systems. The movement of MNPs inside a body is still under question, since there is no absolute knowledge of whether the particles move inside blood vessels or cut through the tissue, therefore damaging the tissue. Such systems may also be forming larger groups, as the magnetic field is indiscriminate; therefore, smaller MNPs can build up and block blood vessels, which can damage the tissue as well, especially with a stronger magnetic field. In addition, scalable production is needed in order to produce bulk MNPs with desirable characteristics, as their shape, size, surface, and morphology are critical to their properties and usability. The unique properties of MNPs benefit immensely to the nanostructured bio-composites used in biomedical applications, since functionalization and modification of MNPs widen their potential use. Despite numerous strategies and approaches in synthetic routes, there remains a need for precise fabrication protocols in order to control their size, shape, and surface morphology, which is needed for clinical applications. However, functionalized and modified MNPs have shown great and promising potential for diagnostics, therapeutics, as well as theranostics. In the future, even more precise and need-oriented MNPs will be developed and established with improved modification that will produce multifunctionalized bio-based composites for various biomedical applications. 

## 7. Conclusions

Several attempts have already been made to increase the efficiency of MNP-based therapies. However, further investigation is required to increase the safety and effectiveness of MNP-based treatments. Nanomaterials based on biodegradable inorganic biomaterials have unique advantages, such as excellent biodegradability, biocompatibility, high pH-responsive drug release, and high capacity of drug loading. Such functionalized MNPs have attracted immense interest in the field of targeted drug delivery. Due to their large surface areas and size-to-volume ratios, they have incredible catalytic properties, which allows them to be implemented in many applications as well as be used in remediation. Despite their smaller sizes, which present a challenge in terms of toxicity, stability, and recovery, surface functionalization and modification of MNPs offers many opportunities for preventing some of these limitations. With surface functionalization and modification, the activity may alter, but still leaves plenty of room for improvement and development of highly active and functional carriers for drug conjugation. At the same time, their colloidal stability, biocompatibility, biodegradability, non-toxicity, and magnetic separability are enhanced, as well as their physical characteristics and chemical functionality. This makes them an excellent tool for improving many fields of biomedicine, especially in therapeutics (drug delivery and hyperthermia) and diagnostics (MRI). However, their coating material must be chosen to ensure the desired characteristics, which are specific to a certain biomedical application. Additionally, multicomponent nanosystems are emerging as theranostic agents, which combine the excellent properties of MNPs and metallic NPs. Such nanomaterials have huge potential in biocatalysis, biosensing, bioimaging, and many other technologies. In order to improve understanding and usage of MNP-based therapies, easy scale-up synthesis methods for MNPs must be established with standardized protocols. On this basis, characterization of multifunctional bio-composites is allowed, i.e., designing efficient magnetic nanocarriers that can meet the requirements for biomedical applications. Tremendous progress was made in the field of biomedical applications for MNPs with in vivo toxicity and their bioprocesses, while targeting the desired location with drug-loaded functionalized MNPs. However, to fully comprehend their potential in targeted delivery, more research must be performed to investigate controlled drug release and therapy to develop an incredible platform for personalized medicine.

## Figures and Tables

**Table 1:** Main advantages and disadvantages of different physical methods for MNP synthesis.

Methods	Advantages	Disadvantages	References
Physical	Higher yields Shorter processing time Simple and easy process No hazardous materials	Lower purity High energy consumption Oxidation of NPs Limited particle control over size, distribution, and morphology	[43,45,46,47,48]
Ball milling	Simple and convenient Top–down approach Formation of the product can be optimized	Contamination of the product Wide size distribution	[49,50,51,52]
Laser ablation	Simple technique Bottom–up approach Low-cost Effective Does not require hazardous materials	Limited size control High energy consumption	[53,54,55,56,57]
Electron beam lithography	High resolution High reliability in processing High accuracy in positioning/alignment High flexibility in pattern replication	High maintenance cost Complicated	[58,59,60,61,62,63]

**Table 2:** Detailed advantages and disadvantages of MNPs prepared by different chemical methods.

Methods	Advantages	Disadvantages	Reference
Chemical (in general)	Lower reaction times Lower reaction temperatures Relative simplicity Use of surfactants for size control and distribution Core shell morphology quality control.	Use of hazardous materials Toxic by-products Large amount of liquid required in the synthesis process Oxidation of NPs aggregation of NPs	[82,83,84,85,86,87,88]
Co- precipitation	High product yield High product purity Narrow size distributions Easily reproducible Environmentally friendly No organic solvents Low cost	MNPs contain large amounts of water Low removal efficiency	[21,87,88,89,90]
Thermal decomposition	Easy method Innovative method Enough thermal energy Produces monodispersed MNPs	Expensive High-temperature requirements Poor dispersions in water	[91,92,93,94,95,96]
Microemulsion	Easy preparation Thermodynamic stability Nano-sized Minimal aggregation Crystalline structure High specific surface area Composition control	Large amounts of surfactants or cosurfactants pH/temp influence the stability	[71,97,98]
Sol–gel method	Homogenous final products High purity Synthesis at low temperature Chemical composition control	Longer reaction times Use of organic solvents	[99,100,101,102,103]
Hydrothermal method	Easy experimental set-up High yields Minimal loss of material Well-controlled material composition	Expensive equipment High temperatures	[104,105,106,107,108,109]

**Table 3:** Advantages and disadvantages of biological methods.

Methods	Advantages	Disadvantages	Reference
Biological	Somewhat monodispersed Manipulation of size and shape by controlling the growth rate of microorganisms Lower toxicity Lower agglomeration Environmentally friendly approaches	Slow and low productivity Wider size distributions Heterogenous sizes and shapes Difficult MNPs and plant separation.	[115,116,117,118,119,120,121,122]

**Table 4:** MNPs used for drug delivery of DOX (conjugated via a noncovalent approach).

Polymer Based MNPs	Drug	Targeting Pathway	Tumour Type Cancer	Conjugation Protocol	Reference
PEGylated	Dox	Passive	Prostate	adsorption	[227]
PVP-stabilized	Dox	Passive	Breast	adsorption	[228]
MNPs	Dox	Active	Breast	adsorption	[229]
SPION	Dox	Passive/Active	Lung	electrostatic interactions	[230]
Dextran	Dox	Passive/Magnetic drug targeting	Liver	adsorption	[231]
PEG	Dox	Passive	Breast	encapsulation	[232]

**Table 5:** MNPs used for drug delivery of DOX conjugated via covalent approach.

**Polymer-Based MNPs**	**Drug**	**Targeting** **Pathway**	**Tumor** **Type Models**	**Conjugation** **Approach**	**Reference**
Dox-hyaluronic acid	DOX	active	ovarian	amide bond	[234]
DOX-ADH	DOX	passive	cancer cells	hydrazone bond	[235]
DOX-PEG	DOX	passive/magnetic drug targeting	tumor cells	hydrazone bond	[236]
DOX	DOX	active/magnetic drug targeting	cancer cells	hydrazone bond	[237]

## Data Availability

The raw data supporting the conclusions of this article will be made available by the authors upon request.
